# A phase 1 study of biweekly nab-paclitaxel/oxaliplatin/S-1/LV for advanced upper gastrointestinal cancers: TCOG T1216 study

**DOI:** 10.1093/oncolo/oyae109

**Published:** 2024-06-21

**Authors:** Hui-Jen Tsai, Shih-Hung Yang, Chin-Fu Hsiao, Hsiang-Fong Kao, Yung-Yeh Su, Yan-Shen Shan, Chia-Jui Yen, Jeng-Shiun Du, Chiun Hsu, I-Chen Wu, Li-Tzong Chen

**Affiliations:** National Institute of Cancer Research, National Health Research Institutes, Tainan, Taiwan; Department of Oncology, National Cheng Kung University Hospital, College of Medicine, National Cheng Kung University, Tainan, Taiwan; Division of Hematology and Oncology, Department of Internal Medicine, Kaohsiung Medical University Hospital, Kaohsiung Medical University, Kaohsiung, Taiwan; Department of Oncology, National Taiwan University Hospital, Taipei, Taiwan; Institute of Population Health Sciences, National Health Research Institutes, Zhunan, Taiwan; Department of Oncology, National Taiwan University Hospital, Taipei, Taiwan; National Taiwan University Cancer Center, Taipei, Taiwan; National Institute of Cancer Research, National Health Research Institutes, Tainan, Taiwan; Department of Oncology, National Cheng Kung University Hospital, College of Medicine, National Cheng Kung University, Tainan, Taiwan; Division of Hematology and Oncology, Department of Internal Medicine, Kaohsiung Medical University Hospital, Kaohsiung Medical University, Kaohsiung, Taiwan; Center for Cancer Research, Kaohsiung Medical University, Kaohsiung, Taiwan; Department of Surgery, National Cheng Kung University Hospital, College of Medicine, National Cheng Kung University, Tainan, Taiwan; Department of Oncology, National Cheng Kung University Hospital, College of Medicine, National Cheng Kung University, Tainan, Taiwan; Division of Hematology and Oncology, Department of Internal Medicine, Kaohsiung Medical University Hospital, Kaohsiung Medical University, Kaohsiung, Taiwan; Department of Oncology, National Taiwan University Hospital, Taipei, Taiwan; Center for Cancer Research, Kaohsiung Medical University, Kaohsiung, Taiwan; Division of Gastroenterology, Department of Internal Medicine, Kaohsiung Medical University Hospital, Kaohsiung Medical University, Kaohsiung, Taiwan; National Institute of Cancer Research, National Health Research Institutes, Tainan, Taiwan; Department of Oncology, National Cheng Kung University Hospital, College of Medicine, National Cheng Kung University, Tainan, Taiwan; Center for Cancer Research, Kaohsiung Medical University, Kaohsiung, Taiwan; Division of Gastroenterology, Department of Internal Medicine, Kaohsiung Medical University Hospital, Kaohsiung Medical University, Kaohsiung, Taiwan

**Keywords:** phase 1, nab-paclitaxel, oxaliplatin, S-1/leucovorin, upper gastrointestinal cancers

## Abstract

**Background:**

Oxaliplatin- and fluoropyrimidine-based triplet regimens have demonstrated feasibility and efficacy in the treatment of upper gastrointestinal (UGI) cancers. Herein, we evaluate the feasibility and preliminary efficacy of biweekly nab-paclitaxel plus oxaliplatin and S-1/leucovorin (SOLAR) in chemonaïve UGI cancers.

**Methods:**

A 3 + 3 phase 1 study was conducted to determine the maximal tolerated dose (MTD) of oxaliplatin in SOLAR (nab-paclitaxel [150 mg/m^2^ in D1], oxaliplatin [60, 75, or 85 mg/m^2^ in D1], and oral S-1/leucovorin [35 mg/m^2^ and 30 mg bid from D1 to D7]). The secondary endpoints were overall response rate (ORR), progression-free survival (PFS), overall survival (OS), and safety.

**Results:**

Thirteen and 6 accruals were in the dose-escalation and MTD expansion cohorts, respectively. One of 6 patients at level III experienced dose-limiting toxicity (grade 3 diarrhea), which revealed that the MTD of oxaliplatin was 85 mg/m^2^. After a mean of 15.9 cycles of treatment, the most common treatment-related grade 3/4 toxicities were neutropenia (57.9%) and diarrhea (21.1%). The ORR was 63.2%. The median PFS and OS were 12.5 and 24.7 months, respectively.

**Conclusion:**

The current study revealed the MTD of oxaliplatin and demonstrated the preliminary efficacy of SOLAR in UGI cancers, which deserves further investigation.

**ClinicalTrials.gov Identifier:**

NCT03162510

## Teaser

The results of this study revealed the maximal tolerated dose of oxaliplatin and preliminary efficacy of SOLAR in upper gastrointestinal cancers, which deserves further investigation.

**Lessons Learned**

- Replacement of 48-hour infusion of 5-FU/LV with oral S-1/LV improves the convenience of treatment administration.
- The maximal tolerated dose of oxaliplatin was determined to be dose level 3 (85 mg/m^2^) when combined with nab-placlitaxel (150 mg/m^2^) and S-1 (35 mg/m^2^ bid)/LV (30 mg bid).
- Triplet combination of biweekly nab-paclitaxel, oxaliplatin, and S-1/LV has manageable toxicities and promising efficacy.

## Discussion

The current phase 1 trial defined the MTD of oxaliplatin to be 85 mg/m^2^ of a biweekly triplet nab-paclitaxel plus oxaliplatin and oral S-1/leucovorin (LV) regimen for UGI cancers (gastric cancer [GC], pancreatic cancer [PC], and biliary tract cancer [BTC]).

Thirteen patients were in the dose-escalation cohort. One patient at dose level III (oxaliplatin 85 mg/m^2^) incidentally received 60 mg/m^2^; therefore, the number of patients with 60, 75, and 85 mg/m^2^ oxaliplatin was 4, 3, and 6, respectively. No dose-limiting toxicity (DLT) was observed in patients at dose levels I and II. One of the 6 patients at dose level III developed DLT (grade 3 diarrhea). Six additional patients were enrolled in the expansion cohort at the MTD. The mean treatment cycles of all patients were 15.9 (3-57) with 16.3, 9.3, and 17.4 cycles for oxaliplatin dose levels of 60, 75, and 85 mg/m^2^, respectively. The most common treatment-related grade 3/4 toxicities were neutropenia (57.9%) and diarrhea (21.1%), which were comparable to those of mFOLFIRINOX in Asian patients with PC.^1^ In our study, the mean dose intensities of all 3 drugs in MTD cohort, were above 80%. Besides, most patients developing grade 3/4 diarrhea were tolerable after dose reduction of S-1. Therefore, SOLAR regimen was tolerable for most patients.

**Figure 1.**
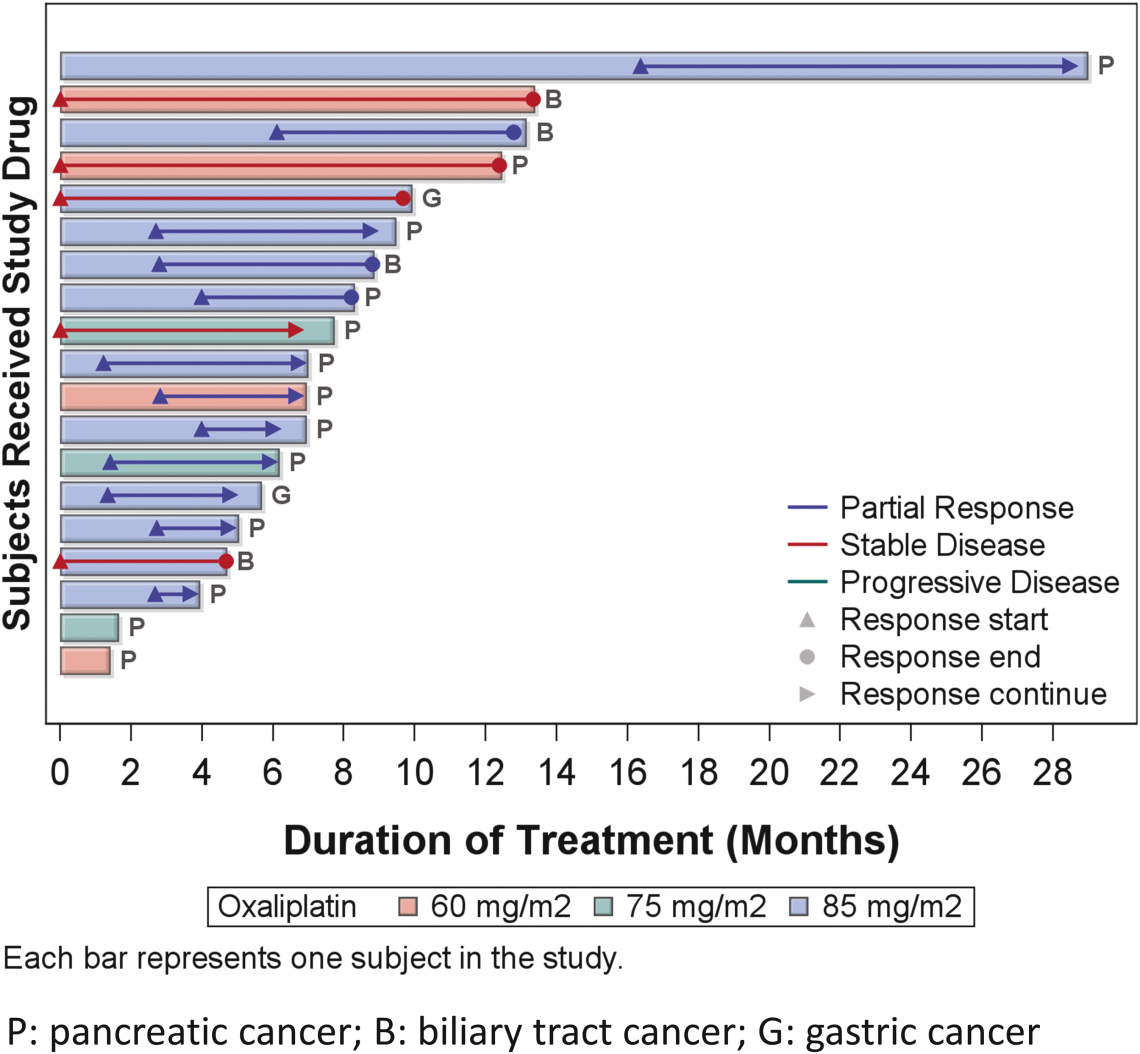
The treatment response, treatment duration, and response duration of each patient.

Regarding the efficacy of SOLAR regimen (Figure 1), the best tumor response of all intention-to-treat patients was partial response (PR) in 12 (63.2%) patients, stable disease (SD) in 5 (26.3%) patients, and progressive disease (PD) in 2 (10.5%) patients. The ORRs in 2 GC, 13 PC, and 4 BTC patients were 50%, 69.2%, and 50%, respectively. Of the 12 patients who received oxaliplatin at an 85 mg/m^2^ dose level, the ORRs of 7 PC, 2 GC, and 3 BTC were 100%, 50%, and 66.7%, respectively. Four patients with metastatic PC had explorative laparotomy after a median of 16.5 cycles of SOLAR regimen. The OS of these 4 patients were 17.1, 17.7, 30.0, and 40.7 months, respectively, and were still alive at the cutoff date of April 30, 2022. Among the 13 patients with PC, the ORR was comparable to 71% in a phase 1b/2 study using nab-paclitaxel plus gemcitabine plus cisplatin (NABPLAGEM) and higher than the other triplet regimens in phase 2 and 3 trials, including FOLFOX plus nab-paclitaxel (FOLFOX-A), and liposomal irinotecan plus oxaliplatin plus 5-FU/LV (NALIRIFOX).^2-4^ Moreover, the median PFS and OS were longer than other doublet or triplet regimens. The efficacy of SOLAR regimen to GC and BTC was comparable with other combinational chemotherapies although the case number is limited.

This study demonstrated acceptable toxicities and a durable response for advanced UGI cancers. Further phase 2 studies are warranted to evaluate the efficacy.

**Table:** 

Trial Information	
Disease	Gastric, pancreatic and biliary tract cancer
Stage of disease/treatment	Advanced/combinational chemotherapy
Prior therapy	None
Type of study	Phase 1, 3 + 3
Primary endpoint	The maximal tolerated dose of oxaliplatin in the triplet chemotherapy (nab-paclitaxel plus oxaliplatin plus S-1/LV)
Secondary endpoints	Overall response rate (ORR), progression-free survival (PFS), overall survival (OS) and safety

## Additional details of endpoints or study design

This study was an open-label phase 1 dose-escalating study and was conducted in 3 institutions in Taiwan. The study was approved by the institutional review board of each participating institution and was performed in accordance with the guidelines and regulations of each participating hospital. Written informed consent was obtained from all participating patients. This trial was registered with Clinical Trials.gov, NCT03162510.

The primary objective was to determine the maximal tolerated dose (MTD) of oxaliplatin in this combination regimen. The secondary objectives were overall response rate (ORR), progression-free survival (PFS), overall survival (OS), and safety.

**Table:** 

Drug Information	
Generic/working name	Oxaliplatin/Oxalip
Company name	TTY Biopharm Co., Ltd.
Drug type	Platinum with antineoplastic activity
Drug class	Platinum compound
Dose	60-85 mg/m^2^
Route	IV
Schedule of administration	Day1 every 2 weeks
Drug Information	
Generic/working name	Nab-paclitaxel/abraxane
Company name	Celgene Ltd
Drug type	Nanoalbumin-conjugated paclitaxel
Drug class	Taxane compound
Dose	150 mg/m^2^
Unit	
Route	IV
Schedule of administration	Day1 every 2 weeks
Drug Information	
Generic/working name	Tegafur, 5-chloro-2,4-dihydroxypyridine and potassium oxonate**/**S-1
Company name	TTY Biopharm Co., Ltd.
Drug type	Pyrimidine fluoride-derived anticancer agent
Drug class	Fluoropyrimidine compound
Dose	35 mg/m^2^ bid
Unit	
Route	Oral
Schedule of administration	Day1 to day 7 every 2 weeks
Drug Information	
Generic/working name	Calcium folinate/Leucovorin
Company name	TTY Biopharm Co., Ltd.
Drug type	Folinic acid
Drug class	Folinic acid
Dose	30 mg bid
Unit	
Route	Oral
Schedule of administration	Day 1 to day 7 every 2 weeks

## Additional details of patient eligibility and rationale for this phase 1 study

The inclusion criteria of this study included

1. pathologically confirmed adenocarcinoma or carcinoma of the stomach, pancreas, and biliary tract with unresectable, recurrence, or metastatic disease;
2. no prior systemic chemotherapy except adjuvant chemotherapy completed 6 months prior enrollment;
3. palliative radiation therapy to bone but not the primary, main tumor site is permitted;
4. at least one measurable lesion over a nonradiated site;
5. age between 20 and 70 years old;
6. Eastern Cooperative Oncology Group (ECOG) Performance Status ≤ 1;
7. life expectancy greater than 12 weeks;
8. adequate bone marrow function: absolutely neutrophil count ≥ 1.5 × 10^9^/L or WBC ≥ 4 × 10^9^/L; hemoglobin ≥ 9 g/dL; platelet count ≥ 100 × 10^9^/L;
9. adequate liver function: ALT ≤ 2.5 × upper limit of normal (ULN) and bilirubin < 1.5 × ULN; and
10. adequate renal function: creatinine < 1.5 × ULN and calculated eGFR > 50 mL/minute.

The exclusion criteria of this study included

1. major surgery within 4 weeks prior to study enrollment;
2. patient with Ampulla vater cancer is excluded;
3. patients with suspicious or history of central nervous system metastasis;
4. patients who with active or uncontrolled infection;
5. patients who have history of myocardial infarction or unstable angina within 6 months before entry;
6. patients with concomitant illness that might be aggravated by chemotherapy;
7. patients who are pregnant or with breast feeding;
8. other concomitant or previously malignancy within 3 years except for in situ cervix cancer or squamous cell carcinoma of the skin, stage 0-1 colon or breast treated by surgery only and without evidence of relapsed tumor;
9. mental status is not fit for clinical trial; and
10. fertile men and women unless using a reliable and appropriate contraceptive method.

Patients with advanced gastric, pancreatic, and biliary tract cancer have a poor prognosis. Platinum (cisplatin or oxaliplatin) plus fluoropyrimidine (capecitabine or fluorouracil [5-FU]) is a common backbone of first-line treatment regimens for advanced gastric and pancreatic cancers and shows the activity as second-line treatment in biliary tract cancer (BTC).^2-8^ Recently, oxaliplatin plus leucovorin (LV)-modulated infusion 5-FU-based triplet chemotherapy regimens have become options for standard therapy in certain upper gastrointestinal (UGI) cancers, such as FOLFIRINOX (irinotecan, oxaliplatin, and 5-FU/LV) as first-line treatment for metastatic pancreatic cancer and perioperative FLOT (docetaxel, oxaliplatin, and 5-FU/LV) for locally advanced gastric cancer (GC) and second-line FOLFOX for gemcitabine and cisplatin-failed BTC.^5,9,10^ However, both triplet regimens are associated with significant hematological adverse events, notably in Asian populations. Development of more tolerable, effective regimens is warranted to improve the therapeutic outcomes of patients with advanced, metastatic UGI cancers. Nab-paclitaxel, a new formulation of nanoalbumin-conjugated paclitaxel, has demonstrated activity either in combination with gemcitabine against gemcitabine alone for patients with metastatic PC or as monotherapy versus solvent-based paclitaxel in previously treated patients with advanced GC.^11,12^ Recently, Bachet et al^13^ showed nab-paclitaxel plus simplified 5-FU/LV infusion every 2 weeks could achieve a similar 4-month PFS rate and different toxicity profile compared to standard nab-paclitaxel and gemcitabine combination in a randomized phase 2, AFUGEM GERCOR trial. Whiles, Safran et al^14^ subsequently demonstrated the feasibility and efficacy of nab-paclitaxel, oxaliplatin and 5-FU/LV combination every 2 weeks, the FOLFOX-A regimen, for patients with advanced PC. S-1, an oral fluoropyrimidine-based agent designed to maintain high levels of 5-FU in plasma and tumor cells and to reduce gastrointestinal toxicity,^15^ has shown moderate single agent activity in GC, PC, and BTC.^16-20^ The safety and efficacy of combining oxaliplatin with oral S-1/LV (1 week on/1 week off), the SOL regimen, has been well established in phase 2/3 trials for colorectal and gastric cancers.^18,21^ Replacing the 48-hour infusional 5-FU/LV in the GOFL (gemcitabine + oxaliplatin + 5-FU/LV) regimen with oral S-1/LV, the SLOG (S-1/LV + oxaliplatin + gemcitabine) regimen, not only improved the convenience of treatment administration but also enhanced therapeutic efficacies for advanced PC.^19,22^

Based on the exciting 60% objective response rate of FOLFOX-A, we designed a phase 1 study to evaluate the feasibility and preliminary efficacy of a nab-paclitaxel, oxaliplatin, and oral S-1/LV combination, the SOLAR regimen, in patients with chemonaïve, advanced UGI cancers. With the known overlapping peripheral neurotoxicity of nab-paclitaxel and oxaliplatin, we firstly fixed the dose of nab-paclitaxel at 150 mg/m^2^ and replaced simplified infusion 5-FU/LV with oral S-1 and LV at 35 mg/m^2^ and 30 mg twice daily, as determined in phase 1 trial of FOLFOX-A and SLOG, respectively, to combining with an escalating dose of oxaliplatin from 60, 75 to 85 mg/m^2^.^14,19^

**Table:** 

Patient Characteristics	
Number of patients	
Male	12
Female	7
Stage	
Locally advanced	4
Metastatic	15
Age: median (range)	57 (29-68)
Number of prior systemic therapies: median	0
Performance status: ECOG	
0	10
1	9
2	0
3	0
4	0
Cancer types or histologic subtypes	
Gastric cancer	2
Pancreatic cancer	13
Biliary tract cancer	4

**Table:** 

Primary Assessment Method	
Title	Dose-limiting toxicity
Number of patients screened	19
Number of patients enrolled	19
Number of patients evaluable for toxicity	19
Number of patients evaluated for efficacy	19
Evaluation method	National Cancer Institute Common Terminology Criteria for Adverse Events version 4.0
Outcome notes	Of the 13 in the dose-escalation cohort, one patient at dose level III (oxaliplatin 85 mg/m^2^) incidentally received 60 mg/m^2^; therefore, the number of patients with 60, 75, and 85 mg/m^2^ oxaliplatin was 4, 3, and 6, respectively. No DLT was observed in patients at dose levels I and II. One of the 6 patients at dose level III developed DLT (grade 3 diarrhea). The MTD of oxaliplatin was determined to be 85 mg/m^2^. To further explore the preliminary efficacy of the regimen, 6 additional patients were enrolled in the expansion cohort at the MTD. The causes of treatment discontinuation were disease progression in 9 patients, treatment-related adverse events (TRAEs) in 5 patients, further treatment plan, conversion surgery, or local ablation therapy, by investigator’s decision in 5 patients. The median time to treatment failure was 7.0 months (95% CI, 5.0-9.5). The mean treatment cycles of all patients were 15.9 (3-57) with 16.3, 9.3, and 17.4 cycles for oxaliplatin dose levels of 60, 75, and 85 mg/m^2^, respectively. Among the 12 patients who received the MTD of oxaliplatin at 85 mg/m^2^, the mean relative dose intensities of nab-paclitaxel, S-1, and oxaliplatin per cycle were 83.1%, 84%, and 83.2%, respectively. (See also Tables 1–3 and Figures 2 and 3).

**Table:** 

Secondary Assessment Method	
Title	Efficacy
Number of patients screened	19
Number of patients enrolled	19
Number of patients evaluable for toxicity	19
Number of patients evaluated for efficacy	19
Evaluation method	RECIST 1.1
Response assessment, CR	0 (0%)
Response assessment, PR	12 (63.2%)
Response assessment, SD	5 (26.3%)
Response assessment, PD	2 (10.5%)
Median duration assessments, PFS	12.5 months (95% CI, 8.3-13.3)
Median duration assessments, TTP	7.0 months (95% CI, 5.0-9.5)
Median duration assessments, OS	24.7 months (95% CI, 17.6-34.5)

## Assessment, analysis, and discussion

**Table:** 

Completion	Study completed
Investigator’s assessment	Active and should be pursued further

Patients with advanced gastric, pancreatic, and biliary tract cancer have a poor prognosis. Platinum, fluoropyrimidine, taxane, gemcitabine, and irinotecan are common chemotherapeutic agents used for the treatment of these cancers.^2,5-9,11,17,19,23-28^ Double or triple chemotherapy regimens have been evaluated to prolong the survival of the patients. Many novel agents and combinational regimens are still under investigation for their safety and efficacy to these devastating diseases.

The combination of 2 chemotherapeutic agents, such as S-1 ± LV plus oxaliplatin, S-1 plus cisplatin, nab-paclitaxel plus S-1, and nab-paclitaxel plus gemcitabine, or 3 chemotherapeutic agents, such as FLOT, NALIRIFOX, S-1/LV plus oxaliplatin and gemcitabine (SLOG), and nab-paclitaxel plus gemcitabine and cisplatin has been studied in patients with advanced UGI cancer.^2,4,5,7,9,11,17,19,24^ The common grade 3/4 treatment-related toxicities of doublet chemotherapeutic agents were neutropenia (15%-38%), diarrhea (5.6%-17%), stomatitis (1.5%-13%), anorexia (15.4%-30%), and peripheral sensory neuropathy (4.7%-17%),^7,11,17,24^ whereas the incidence of grade 3/4 neutropenia was significantly increased using triplet drugs with FLOT (51%), FOLFIRINOX (45.7%), and SLOG (40.7%).^5,9,19^ In the Japanese population, a higher incidence of grade 3/4 neutropenia was noted in patients with PC with FOLFIRINOX (77.8%) and nab-paclitaxel plus gemcitabine (70.6%), and in patients with advanced BTC with gemcitabine/cisplatin (48%).^1,29-31^ The results demonstrated a higher incidence of toxicities by either doublet or triplet chemotherapeutic agents in the Asian population. Of the 12 patients who received an 85 mg/m^2^ starting dose of oxaliplatin and a median of 17.4 cycles of treatment in our T1216 study, the incidence of grade ≥ 3 neutropenia was 58.3%. However, the mean dose intensities of all 3 drugs, ie, nab-paclitaxel, oxaliplatin, and S-1, were above 80%. The findings indicate that the neutropenia of the SOLAR regimen was manageable. The Brown University Oncology Research Group (BrUOG) has performed a phase 1 study using a combination of oxaliplatin (85 mg/m^2^), LV (400 mg/m^2^), 5-FU (2400 mg/m^2^), and dose-escalated nab-paclitaxel (FOLFOX-A) biweekly for patients with advanced PC. The MTD of nab-paclitaxel was 150 mg/m^2^. The incidences of grade 3/4 neutropenia and neuropathy were 17.1% and 5.7%, respectively.^14^ In their study, grade 3 neuropathy was the most significant toxicity. Therefore, they amended the study to reduce the dose of oxaliplatin when grade 2 neuropathy developed.^14^ In Giommoni’s study, they used nab-paclitaxel to replace irinotecan (NAB-FOLFOX) or oxaliplatin (NAB-FOLFIRI) of FOLFIRINOX for metastatic PC. The incidence of grade 3/4 neutropenia (28.6%) and sensory neuropathy (7.1%) was higher for NAB-FOLFOX.^32^ In our study, peripheral sensory neuropathy was the most common toxicity, but all were grade 1 or 2. Because peripheral neuropathy is a concerning toxicity of both nab-paclitaxel and oxaliplatin, we discontinued the drugs when unrecovered grade 2 neuropathy occurred. Two patients had grade 2 sensory neuropathy and withdrew from the study due to concerns about neuropathy after 14 (oxaliplatin 75 mg/m^2^) and 9 (oxaliplatin 85 mg/m^2^) cycles of the SOLAR regimen. The other patients were tolerable with neuropathy. In our study, the incidence of grade 3/4 diarrhea was 33.3% for the 12 patients receiving the MTD of oxaliplatin, 85 mg/m^2^. This incidence was higher than that reported in other trials. However, most cases were tolerable after dose reduction of S-1. In general, the combination of S-1/LV, nab-paclitaxel, and oxaliplatin was tolerable for patients between 20 and 70 years old.

In the current T1216 study, among the 13 patients with advanced PC, the ORR and disease control rate were 69.2% and 84.6%, respectively. The ORR of SOLAR was comparable to 71% in 25 patients with nab-paclitaxel plus gemcitabine plus cisplatin (NABPLAGEM) in a multicenter phase 1b/2 study.^2^ In addition, it was numerically higher than that of 29% in the 48 patients receiving FOLFOX plus nab-paclitaxel (FOLFOX-A) in the BrUOG-292 study,^3^ 41.8% of liposomal irinotecan, oxaliplatin plus 5-FU/LV (NALIRIFOX) in the randomized phase 3 NAPOLI-3 trial^4^ and 39.7% of sequential nab-paclitaxel and gemcitabine followed by modified FOLFOX6 (nab-P/Gem-mFOLFOX) in the randomized phase 2 SEQUENCE trial.^33^ Disease progression as the cause of treatment discontinuation occurred in 30.8% (4/13), 32.0% (8/25), and 68.4% (52/76) of patients receiving corresponding treatments (SOLAR, NABPLAGEM, and nab-P/Gem-mFOLFOX). Of the remaining 9 patients who were off-treatment for non-PD etiology in the current study, 4 had maintenance gemcitabine plus S-1, and 4 had definitive local therapy (RFA for liver metastases plus concurrent chemoradiation therapy for primary tumor in one and conversion surgery in 3). Those subsequent treatments significantly extended the median PFS and OS of the 13 intention-to-treat patients with PC to 12.4 and 24.7 months, respectively, compared to those of 7.4-7.9 and 11.1-13.2 months in patients receiving nab-P/Gem-mFOLFOX or NALIRIFOX in randomized trials.^4,33^

The median OS for patients with advanced GC who received first-line standard chemotherapy with fluoropyrimidine and platinum-containing regimens was 9.3-14.1 months.^6,7,23^ The combination of nivolumab to S-1 plus oxaliplatin (SOX) or capecitabine plus oxaliplatin (CapOX) has been shown to achieve a higher RR (57.1% and 76.5%) and longer survival as first-line treatment for advanced GC in a phase 2 study (ATTRACTION-4).^34^ The median PFS for nivolumab plus SOX and nivolumab plus CapOX was 9.7 and 10.6 months, respectively. The median OS was not reached.^34^ For advanced BTC, gemcitabine plus cisplatin is the current standard first-line treatment.^35^ Gemcitabine plus S-1 has been shown to be noninferior to gemcitabine plus cisplatin, with a median OS of 15.1 months.^20^ Capecitabine plus oxaliplatin (XELOX) or gemcitabine plus oxaliplatin (GEMOX) has been shown to achieve a median OS of 10.6 and 10.4 months, respectively, as first-line treatment for advanced BTC in a phase 3 study.^25^ Two triplet regimens (oxaliplatin plus irinotecan plus S-1 and gemcitabine plus cisplatin plus nab-paclitaxel) have also been shown to achieve a median OS of 12.5 and 19.5 months as first-line treatment for advanced BTC in phase 2 studies.^26,27^ In another phase 2 study, first-line nab-paclitaxel plus gemcitabine was shown to achieve a median OS of 12.4 months for patients with advanced cholangiocarcinoma.^28^ Although the case number in GC and BTC was limited in our study, the RR and OS were comparable with other combinational therapies.^6,7,20,23,25-28^ Therefore, a phase 2 study to confirm the efficacy of the SOLAR regimen is warranted.

**Figure 2.**
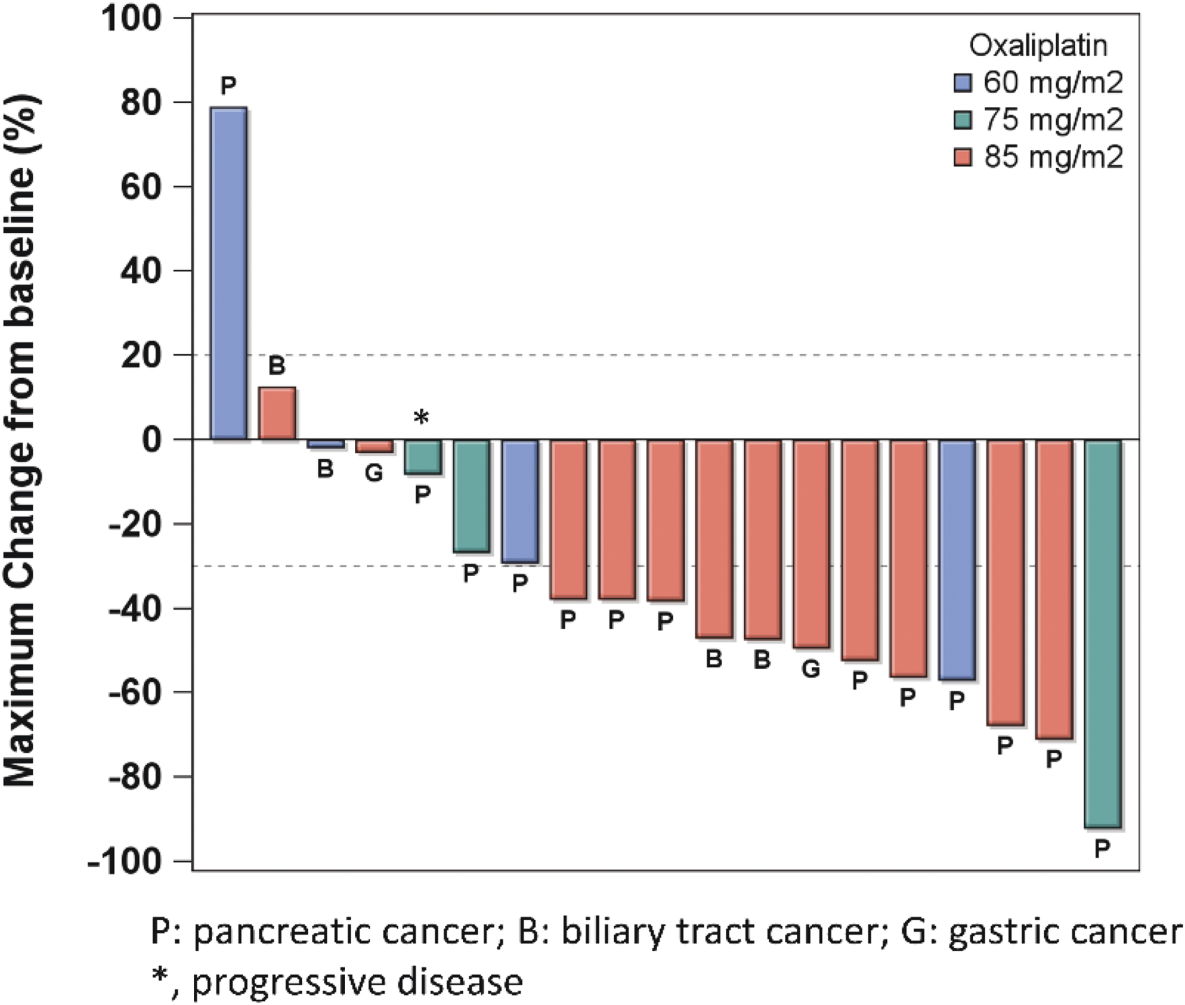
Waterfall plot.

**Figure 3.**
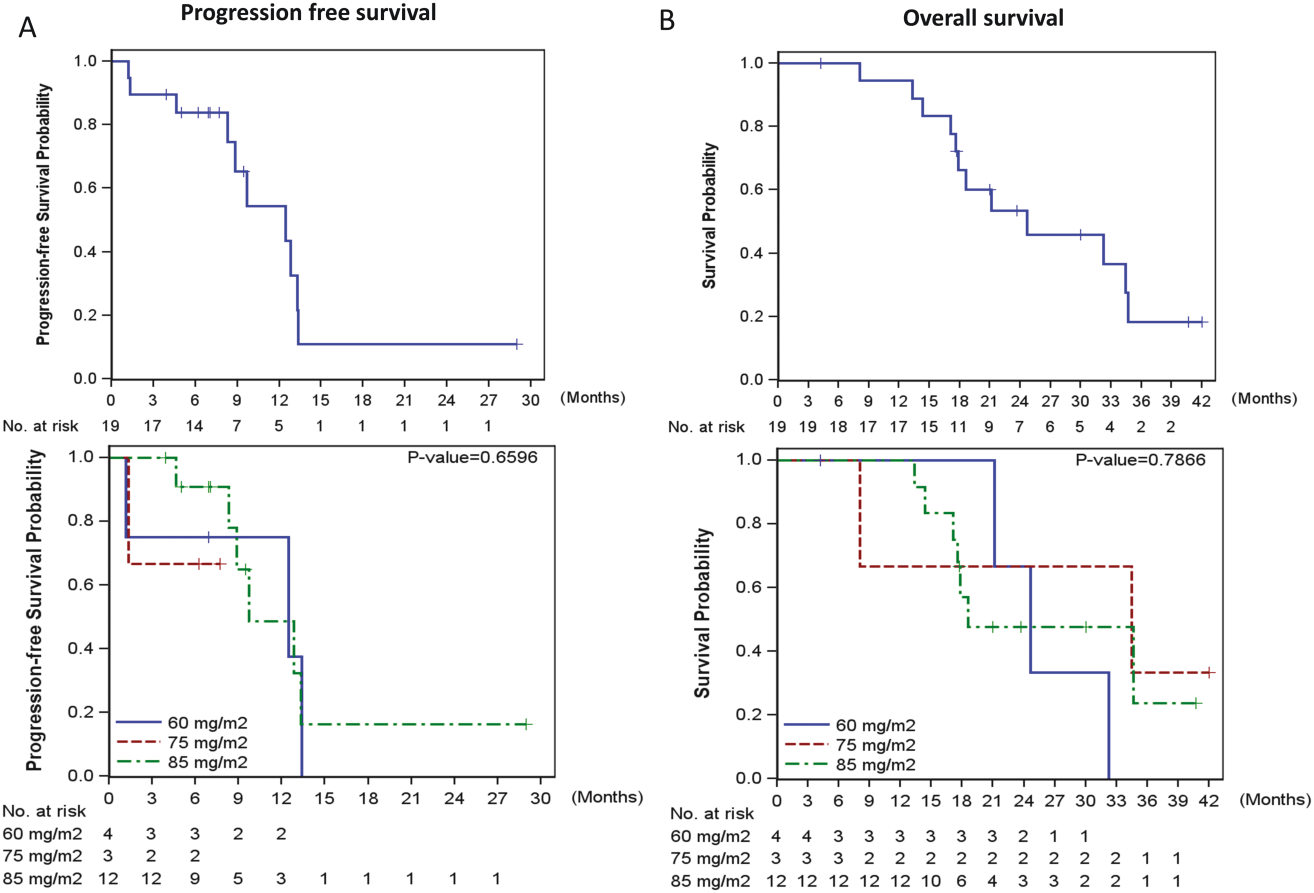
Kaplan-Meier plot.

**Table 1.** Adverse events.

	60 mg/m^2^, *N* = 4		75 mg/m^2^, *N* = 3		85 mg/m^2^, *N* = 12		All	
	All	≥G3	All	≥G3	All	≥G3	All (%)	≥G3 (%)
Neutropenia	2	2	2	2	11	7	15 (78.9)	11 (57.9)
Thrombocytopenia	2	0	2	0	10	1	14 (73.7)	1 (5.3)
Anemia	2	0	2	1	6	0	10 (52.6)	1 (5.3)
Alopecia	3	0	3	0	9	0	15 (78.9)	0
Diarrhea	1	0	1	0	10	4	12 (63.2)	4 (21.1)
Fatigue	2	0	2	0	7	1	11 (57.9)	1 (5.3)
Nausea	2	1	2	0	6	0	10 (52.6)	1 (5.3)
Vomiting	1	0	2	0	7	1	10 (52.6)	1 (5.3)
Anorexia	1	0	2	0	6	1	9 (47.4)	1 (5.3)
Fever	0	0	2	0	4	0	6 (31.6)	0
Weight loss	1	0	1	0	4	0	6 (31.6)	0
Skin hyperpigmentation	1	0	1	0	4	0	6 (31.6)	0
Oral mucositis	3	0	0	0	2	0	5 (26.3)	0
Peripheral sensory neuropathy	3	0	3	0	10	0	16 (84.2)	0
ALT elevation	1	0	1	0	3	0	5 (26.3)	0
AST elevation	1	0	1	0	5	0	7 (36.8)	0
ALK-P elevation	1	0	0	0	4	0	5 (26.3)	0
GGT elevation	2	0	0	0	3	0	5 (26.3)	0

**Table 2.** Dose-escalation table.

Dose Level	Dose of Oxaliplatin
I	60 mg/m^2^
Ia	50 mg/m^2^ (only if dose-limiting toxicity occurs in ≥2 patients at dose level I cohort)
II	75 mg/m^2^
III	85 mg/m^2^

**Table 3.** Dose-limiting toxicity table.

Grade and Toxicity
1.Grade 4 neutropenia (absolute neutrophil count [ANC] < 500/μL) > 4 days duration
2.Complicated grade 3 or higher neutropenia, febrile neutropenia and/or with concurrent infection
3.Grade 3 thrombocytopenia associated with bleeding
4.Failure to recover to ANC ≥ 1500/μL and platelet ≥ 100 000/μL by day 28 after the first or second dose of treatment
5.Any ≥ grade 3 treatment-related nonhematologic toxicity (except for nausea, vomiting, fatigue, and alopecia)
6.Grade 2 neuropathy without recovery to ≤ grade 1 for more than 2 weeks

## Data Availability

All data generated or analyzed during the current study are included in this published article.

## Notes

**Additional information:** Li-Tzong Chen is the principal investigator.
